# “The feasibility of free septal mucosal grafts in preventing postoperative middle meatus synechia after endoscopic sinus surgery”

**DOI:** 10.1038/s41598-026-63253-0

**Published:** 2026-07-23

**Authors:** Ahmed Elgendy, Saad Elzayat, Ali Gamal El-Ouny, Ahmed Morshedy, Sobhy Khaled, Ibrahim Gehad

**Affiliations:** Otolaryngology Department, Kafrelsheikh University hospital, El-giesh street, Kafrelsheikh, 33155 Egypt

**Keywords:** Middle meatal synechiae prevention, Endoscopic sinus surgery, Free septal mucosal graft, Middle turbinate lateralization, Chronic rhinosinusitis, Diseases, Medical research

## Abstract

This study aimed to evaluate the feasibility and safety of using a free septal mucosal graft to prevent middle meatal synechiae following endoscopic sinus surgery (ESS). This prospective observational study was conducted at a tertiary referral center between March 2022 and February 2024. Adult patients with chronic rhinosinusitis without nasal polyps (CRSsNP) who were refractory to medical therapy were included. All patients underwent unilateral ESS followed by placement of a free septal mucosal graft over exposed bone in the middle meatus. Postoperative outcomes were assessed using the Sinonasal Outcome Test-22 (SNOT-22), Lund–Kennedy Endoscopic Score (LKES), and Perioperative Sinus Endoscopy (POSE) score. A total of 116 patients completed the study. Significant improvement in SNOT-22 scores was observed at 6 months postoperatively (*p* < 0.001). Significant improvement was also noted in endoscopic findings (LKES, *p* < 0.001). Stable middle turbinate position without lateralization was achieved in 91 (78%) of cases. Free septal mucosal grafting appears to be a feasible, biocompatible, and technically straightforward method to reduce middle meatal synechiae following ESS.

**Supplementary Information:**

The online version contains supplementary material available at 10.1038/s41598-026-63253-0.

## Introduction

Chronic rhinosinusitis (CRS) is a prevalent inflammatory condition affecting a substantial proportion of the global population and is associated with a significant impact on quality of life^1^. For patients who are refractory to maximal medical therapy, endoscopic sinus surgery (ESS) represents the cornerstone of management, aiming to restore sinus ventilation and mucociliary clearance. ESS has been consistently shown to provide significant and sustained improvement in sinonasal symptoms and patient-reported quality of life^2^.

Despite advances in surgical techniques, postoperative complications such as middle meatal synechiae (MMS)—adhesions between the middle turbinate and the lateral nasal wall—remain a common challenge. These adhesions may compromise sinus drainage, hinder postoperative endoscopic access, and contribute to disease persistence or the need for revision surgery^3^.

The incidence of synechiae following ESS varies widely, with reported rates ranging from 10% to 40%, depending on surgical technique, disease severity, and postoperative care^4^. These adhesions may result in iatrogenic obstruction of the middle meatus, impairing mucociliary clearance and perpetuating chronic inflammation. Various preventive strategies have been described, including meticulous surgical technique. Maintaining mucosal cover and preserving the natural physiological properties of the paranasal sinus membranes are critical for successful healing and for avoiding long-term complications like synechiae formation. Good endoscopic visualization and the use of delicate instruments profoundly enhance the ability to respect and manipulate these structures with minimal structural trauma^5^. Medialization of the middle turbinate, spacers, stents, and steroid-eluting implants. However, these approaches may be associated with increased cost, foreign body reaction, or patient discomfort, and no single method has been universally accepted as the standard of care^6^.

Autologous mucosal grafting has emerged as a promising biological approach aimed at restoring mucosal integrity and reducing granulation tissue formation. Free mucosal grafts, particularly those harvested from the nasal septum, are biocompatible, readily accessible during ESS, and associated with minimal donor site morbidity. In addition, the nasal septal mucosa shares structural and functional similarities with sinonasal mucosa, which may enhance mucosal regeneration^7^.

The use of septal mucosal grafts to cover exposed surfaces is based on promoting rapid re-epithelialization and preventing adhesion between opposing mucosal surfaces during healing, the utilization of autologous endonasal tissue for defect reconstruction and mucosal surfacing has been heavily validated in expanded endoscopic skull base surgeries, consistently demonstrating excellent biocompatibility, high survival rates, and negligible donor-site morbidity Previous studies in skull base surgery have demonstrated the viability of mucosal grafts for mucosal restoration, although in a different surgical context^8,9^. In ESS, however, the application of free mucosal grafts as a preventive strategy for middle meatal synechiae remains limited, with current evidence largely confined to small case series and pilot studies.

Therefore, this prospective observational study was designed to evaluate the feasibility and clinical outcomes of using free septal mucosal grafts in reducing postoperative synechiae formation following ESS.

## Patients and methods

### Study design and setting

This prospective observational study was conducted at the Otorhinolaryngology Department, Kafrelsheikh University Hospital, Egypt, between March 1, 2022, and February 29, 2024. The study protocol was approved by the Institutional Review Board of Kafrelsheikh University (Approval No.: **MKSU-50-21**; Date: **January 1**,** 2022**), and written informed consent was obtained from all participants. **“The protocol number represents the Institutional Review Board’s internal numbering system and does not indicate the year of approval.”**

Diagnosis was established according to the European Position Paper on Rhinosinusitis and Nasal Polyps (EPOS 2020) criteria^10^.

### Participants

Adult patients (18–60 years) diagnosed with chronic rhinosinusitis without nasal polyps (CRSsNP) and refractory to maximal medical therapy were included.

Inclusion criteria included:

- Unilateral disease predominantly affecting the frontal sinus.
- Persistence of symptoms despite appropriate medical treatment for at least 6 months.

Exclusion criteria included:

- Nasal polyps or eosinophilic CRS.
- Prior sinonasal surgery or severe septal deviation.
- Primary ciliary dyskinesia or cystic fibrosis.
- Sinonasal tumors.
- Pregnancy or coagulation disorders.
- Contraindications to general anesthesia or refusal of surgery.

All patients received standard preoperative medical therapy, including intranasal corticosteroids. The use of systemic corticosteroids was documented when applicable.

### Preoperative assessment

All patients underwent comprehensive history-taking and clinical evaluation by the senior author, including nasal endoscopy and computed tomography (CT) of the paranasal sinuses. The Lund–Mackay score was recorded to assess disease burden^11^.

### Surgical technique

All procedures were performed under general anesthesia by two senior surgeons. Endoscopic sinus surgery (ESS) was performed according to disease extent and included frontal sinusotomy **(Draf IIa in most cases)**.

A free septal mucoperiosteal graft was harvested from the posterior nasal septum. The graft was elevated in a mucoperichondrial plane using a Cottle elevator [10] extending anteriorly approximately 2 cm from the posterior border of the vomer, then a vertical incision till the junction of the nasal floor with the nasal septum inferiorly. The incision was then completed posteriorly till the posterior end of the graft. The donor site was left to heal by secondary intention and was monitored during follow-up.

**Fig. 1:**
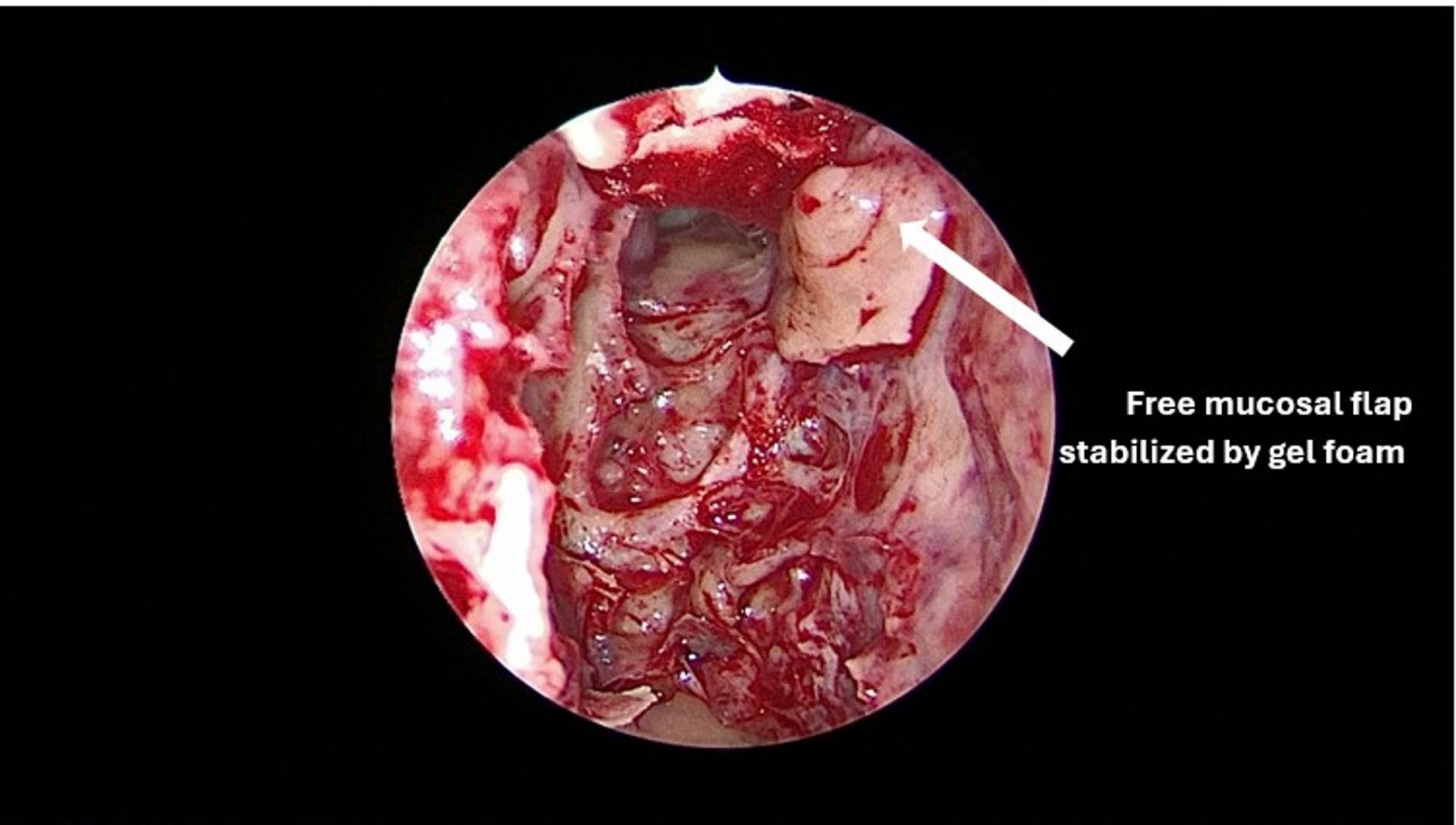
: Free septal mucosal graft situated in the middle meatus after left frontal sinusotomy.

The graft (approximately 1.5 × 1 cm) was positioned with the mucosal surface facing outward to cover exposed bone in the middle meatus adjacent to the middle turbinate. It was secured using gel foam soaked in dexamethasone sodium phosphate solution (concentration: 8 mg/2mL diluted in 10 mL normal saline). **(**Fig. 1**)**

Internal nasal splints *(Medtronic fluoroplastic intranasal splint)* were placed bilaterally and removed after one week. We made sure the splint was lateral to the middle turbinate on the operated site to further prevent lateralization.

### Postoperative care

Postoperative management included:

- Systemic antibiotics for 7 days.
- Nasal saline irrigation three times daily for 1 month.
- Intranasal corticosteroid spray twice daily.

Patient adherence to postoperative treatment was encouraged; however, it was not objectively assessed.

### Outcome measures and follow-up

Patients were followed weekly during the first postoperative month for cleaning, debridement, and nasal endoscopic evaluations (0° and 70°), followed by monthly visits up to 12 months.

Primary outcomes included:

- Feasibility of the technique.
- Incidence of middle meatal synechiae.

Secondary outcomes included:

- Symptomatology was assessed via the validated Arabic version of the Sinonasal Outcome Test-22 (A-SNOT-22) preoperatively, and at one and six months postoperatively^12^.
- Endoscopic findings were scored using the Lund–Kennedy Endoscopic Score^11^.
- The Perioperative Sinus Endoscopy (POSE) scoring system^13^ is at baseline and at one, three, and six months. POSE scoring evaluated frontal sinus/recess and middle turbinate status (0 = healthy; 2 = obstructed/severe synechiae).

Assessments were performed preoperatively and at 1, 3, and 6 months postoperatively.

Graft integration was evaluated endoscopically based on mucosalization, stability, and absence of necrosis.

Documented complications included major events (orbital involvement, cerebrospinal fluid leaks) and minor ones (controlled epistaxis, pain, discharge).

A successful outcome was defined as the absence of middle meatal synechiae and a stable middle turbinate position at 6 months.

### Statistical analysis

Analyses were conducted using IBM SPSS Statistics for Windows, Version 25.0 (IBM Corp., Armonk, NY). Continuous data are presented as mean ± standard deviation (SD) or median with interquartile range (IQR), depending on their distribution; categorical variables are expressed as counts and percentages. The Wilcoxon signed-rank test was applied to pre- versus postoperative comparisons of non-parametric continuous outcomes (e.g., SNOT-22, Lund–Kennedy scores). Statistical significance was set at *p* < 0.05.

## Results

A total of 123 patients were initially enrolled. Seven patients were lost to follow-up, and the final analysis included 116 patients (56 males, 60 females) with a mean age of (36.98 ± 12.17) years old. According to the preoperative CT findings, the Lund-Mackay score ranged from 6 to 12, with a mean of (8.03 ± 1.60). All patients had primary ESS, and none presented with major perioperative complications.

Symptom Outcomes (SNOT-22).

There was a statistically significant improvement in SNOT-22 scores at 6 months postoperatively compared to baseline (median 43 vs. 19, *p* < 0.001). Improvement was observed across all major symptom domains, indicating a substantial reduction in disease burden, as shown in Table 1.

**Table 1:** Shows the results of the SNOT 22 according to Wilcoxon Signed Ranks Test.

	N	Mean	SD	Median	Q1-Q3	P value
SNOT22 (Preoperative)	116	49.32	18.034	43.00	35–61	< 0.001*
SNOT22 (Postoperative)	116	24.92	16.578	19	11.25-41	
SNOT22_olfactory domain (Preoperative)	116	19.42	4.553	18.00	16–21	< 0.001*
SNOT22_olfactory domain (Postoperative)	116	7.64	5.599	6	3–9	
Blow Nose (Preoperative)	116	3.22	1.031	3	3–4	< 0.001*
Blow Nose (Postoperative)	116	1.49	1.293	1	1–2	
Sneezing (Preoperative)	116	3.25	1.029	3	2.5-4	< 0.001*
Sneezing (Postoperative)	116	1.11	1.033	1	0–2	
Runny Nose (Preoperative)	116	3.32	1.068	3	3–4	< 0.001*
Runny Nose (Postoperative)	116	1.35	1.101	1	1–2	
Thick Nasal Discharge (Preoperative)	116	3.22	0.970	3	2.5-4	< 0.001*
Thick Nasal Discharge (Postoperative)	116	1.77	1.359	1	1–2	
Smell/Taste (Preoperative)	116	3.03	1.172	3	2–4	< 0.001*
Smell/Taste (Postoperative)	116	0.52	1.103	0	0.00-0.075	
Blockage/Congestion (Preoperative)	116	3.37	0.983	3	3–4	< 0.001*
Blockage/Congestion (Postoperative)	116	1.83	1.213	1	1–2	

Endoscopic Outcomes (LKES).

Regarding LKES, it was calculated at baseline and at 1, 3, and 6 months postoperatively.

At baseline, the total LKES was (mean 3.97 ± 0.79), with edema and discharge being the most prominent features (means of 1.59 ± 0.51 and 1.66 ± 0.51, respectively).

One month postoperatively, the total LKES slightly increased to (mean 4.43 ± 0.97), primarily due to increased crusting (mean 1.26 ± 0.44) despite marked reductions in polyposis (mean 0.10 ± 0.36).

By the third month, there was a substantial decrease in the total LKES to (mean 2.67 ± 1.04), reflecting continued improvement across most domains, especially crusting (mean 0.23 ± 0.42) and polyposis (mean 0.67 ± 0.52).

At six months, the total LKES remained stable (mean 2.63 ± 1.37), with generally low scores across all domains, although mild increases in synechia (mean 0.29 ± 0.61) which means there are 21.6% of patients with any detectible grade of and discharge (mean 0.90 ± 0.60) were observed.

Finally, there was a significant improvement in LKES at the 6th month compared with the baseline (p-value < 0.001).

**Fig. 2:**
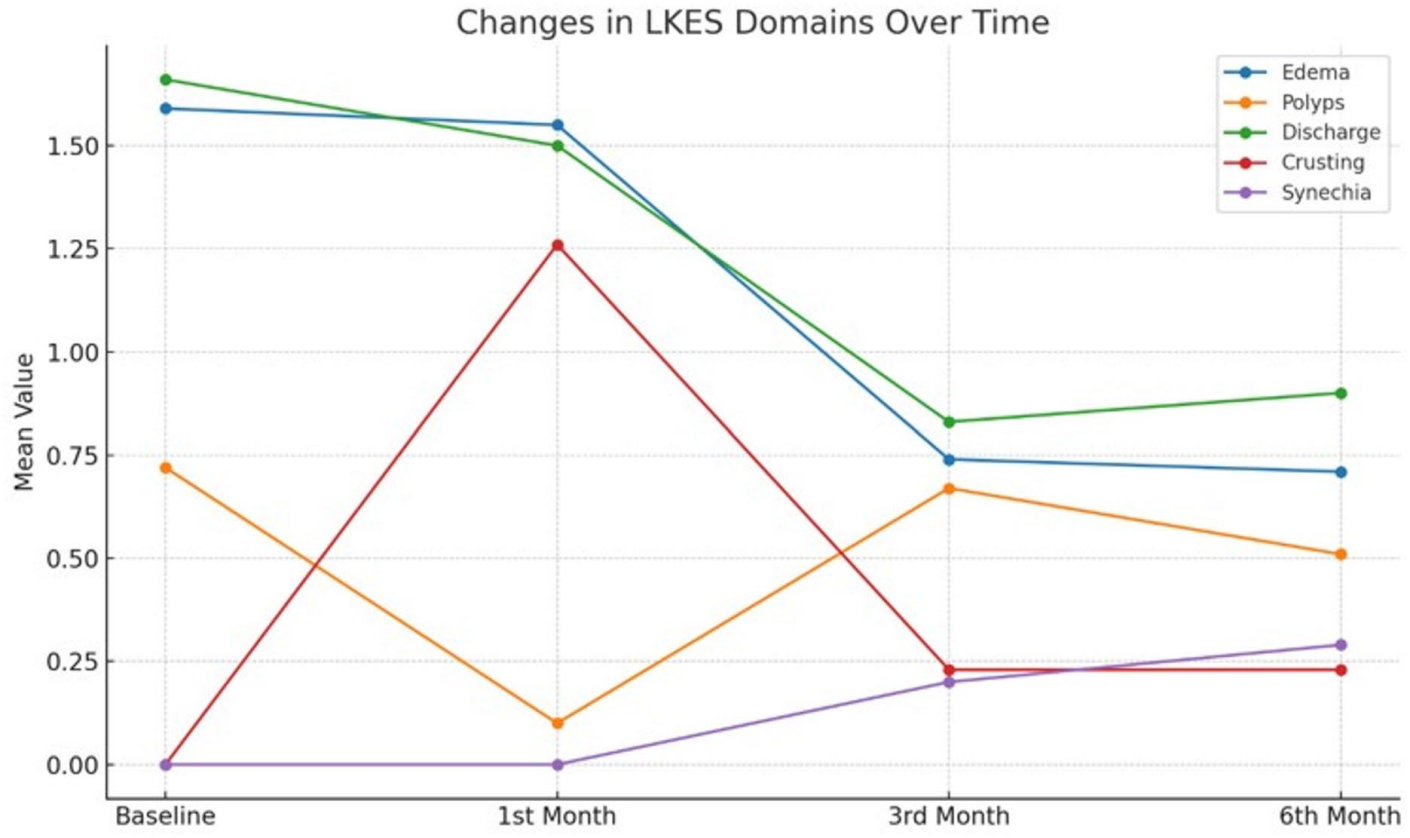
A multiple line graph showing changes of each LKES domain’s mean value over time. (Temporal changes in individual LKES domains over the follow-up period.)

Changes in LKES over the follow-up period are illustrated in Table 2; Fig. 2 demonstrates the temporal improvement in endoscopic findings. A marked reduction in crusting and edema was observed during the early postoperative period, followed by stabilization of mucosal healing at later follow-up intervals.

**Table 2:** LKES Scores (Mean ± SD).

Domain	Baseline (Mean ± SD)	1st Month Post-op (Mean ± SD)	3rd Month Post-op (Mean ± SD)	6th Month Post-op (Mean ± SD)	*P*-value
**Edema**	1.59 ± 0.51	1.55 ± 0.50	0.74 ± 0.51	0.71 ± 0.46	< 0.001*
**Polyps**	0.72 ± 0.76	0.10 ± 0.36	0.67 ± 0.52	0.51 ± 0.54	0.013*
**Discharge**	1.66 ± 0.51	1.50 ± 0.52	0.83 ± 0.55	0.90 ± 0.60	< 0.001*
**Crusting**	0.00 ± 0.00	1.26 ± 0.44	0.23 ± 0.42	0.23 ± 0.42	< 0.001*
**Synechia**	0.00 ± 0.00	0.00 ± 0.00	0.20 ± 0.40	0.29 ± 0.61	< 0.001*
**Total LKES**	3.97 ± 0.79	4.43 ± 0.97	2.67 ± 1.04	2.63 ± 1.37	< 0.001*

P-value for Wilcoxon Signed-Rank Test between 6th Month Post-op Vs baseline scores.

Middle Turbinate Position (POSE Score).

Regarding the middle turbinate’s POSE score at the end of the follow-up period at the 6th month, we had 91 (78.4%) successful cases with no lateralization of the middle turbinate, 17 (14.7%) cases with partial lateralization, and 8 (6.9%) cases with complete lateralization.

This indicates progressive stabilization of the middle turbinate over time.

**Fig. 3:**
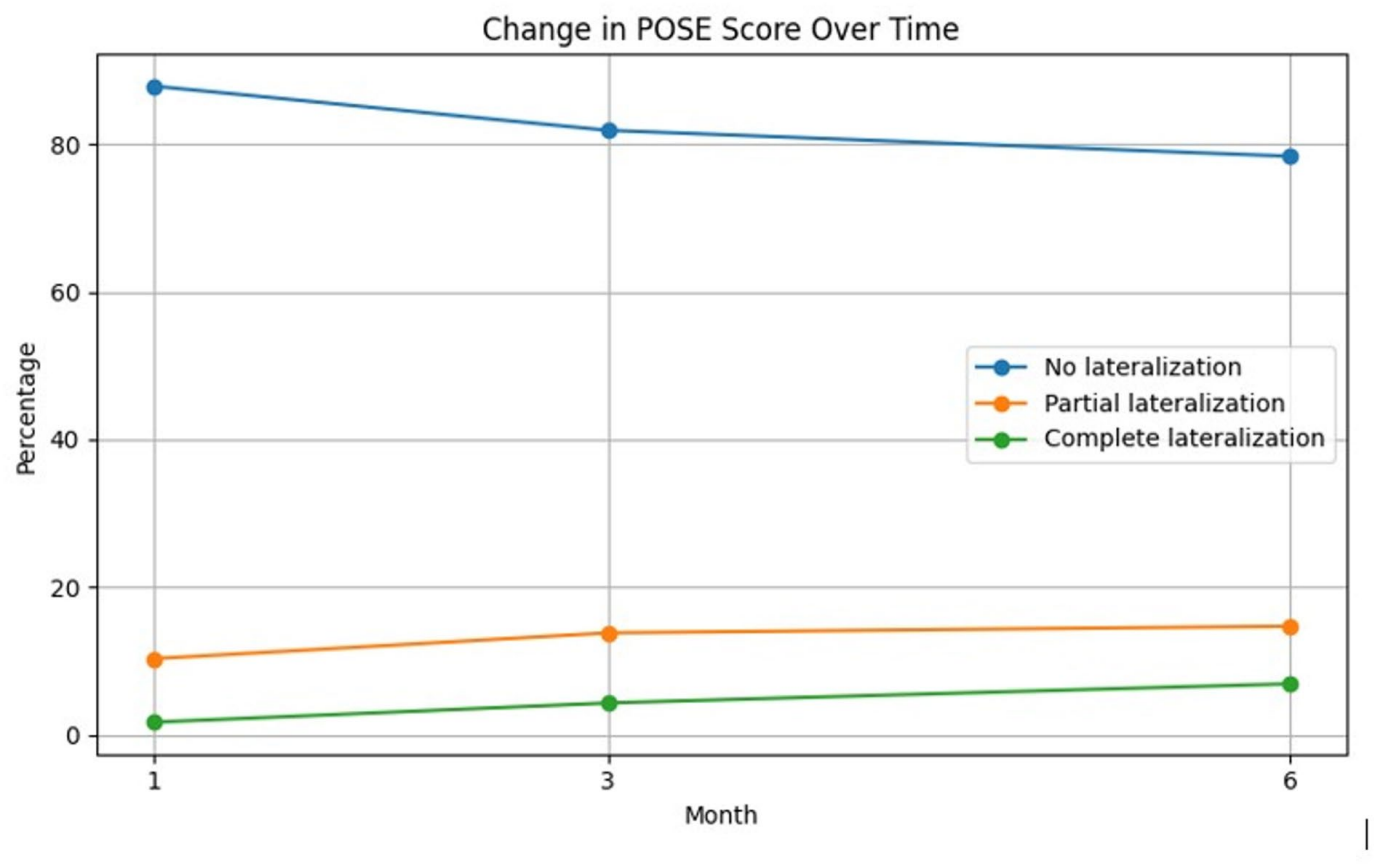
A multiple line graph showing changes of lateralization of middle turbinate over the follow-up period.

Changes in lateralization of middle turbinate during the follow-up period were demonstrated in Fig. 3.

The septal donor site healed satisfactorily in all patients, with no cases of septal perforation or significant complications observed during follow-up.

## Discussion

Endoscopic sinus surgery (ESS) is well established as the standard therapeutic intervention for chronic rhinosinusitis refractory to medical management. However, postoperative adhesion formation remains one of the most common complications, with reported incidence rates ranging from 10% to 40% depending on surgical technique and postoperative care^4^.

Intranasal synechiae result from aberrant mucosal healing following epithelial injury, often induced by surgical manipulation or nasal packing. This process involves disruption of cytokine homeostasis, with fibroblastic and myofibroblastic activity—mediated by transforming growth factor beta (TGF-β)—promoting collagen deposition and adhesion formation^14^.

Middle meatal synechiae are particularly clinically significant, as they may compromise ventilation of the frontal, ethmoid, and maxillary sinuses, impair postoperative endoscopic access, and complicate follow-up care, including routine debridement. Preventing middle meatus synechiae is not merely an anatomical goal; it is clinically imperative to maintain long-term ostial patency. Obstructive scarring and subsequent frontal or ethmoidal sinus outflow tract failure can lead to recurrent disease persistence or, in severe cases, dangerous downstream orbital and intracranial complications requiring hazardous revision interventions^15–17^.

The etiology of synechiae formation after ESS is multifactorial. Contributing factors include mucosal trauma, use of hemostatic agents, inadequate postoperative care, persistent inflammation, extent of surgical dissection, infection, and lateral displacement of the middle turbinate^18,19^. Anatomical instability of the middle turbinate further increases the risk of lateralization and adhesion formation.

Several intraoperative strategies have been proposed to reduce synechiae formation, including middle turbinate medialization, spacers, stents, suture techniques, and topical agents^20–22^. Various packing materials have also been used, ranging from non-absorbable materials such as Merocel to biodegradable alternatives^23,24^. In addition, pharmacological agents such as mitomycin C have demonstrated a potential role in reducing postoperative adhesions^25,26^. Despite these approaches, no single method has been universally accepted as the standard of care.

In the present study, we evaluated a biological approach involving the application of a free septal mucosal graft to the middle meatus. This technique utilizes an autologous, readily accessible donor site with minimal morbidity, allowing intraoperative graft harvesting without prolonging operative time. The graft acts as a biological barrier, covering exposed bone, reducing crust formation, and minimizing the risk of adhesion while preserving sinus patency.

Our findings demonstrate that this technique is feasible and associated with favorable postoperative outcomes, including a low synechiae rate (mean score: 0.29 ± 0.61 at six months) and stable middle turbinate positioning in the majority of patients. Significant improvements were also observed in both endoscopic scores and patient-reported outcomes, suggesting enhanced mucosal healing.

These findings should be interpreted in the context of the study design. As a single-arm prospective observational study, this work primarily demonstrates feasibility and favorable outcomes rather than comparative effectiveness. The absence of a control group limits the ability to attribute outcomes solely to the graft or to establish superiority over other techniques.

When compared with previously reported synechiae rates of approximately 15–30% using middle meatal spacers^20^ and around 21.6% with silver nitrate cauterization^27^, the outcomes observed in this study was also around 21.6% at 6th month; which appear encouraging. However, such comparisons should be interpreted with caution due to differences in study design and patient populations.

In addition to spacers and cauterization techniques, systematic reviews have demonstrated variable outcomes with steroid-eluting implants and absorbable packing materials, with no single technique showing consistent superiority across studies^28^.

The biological advantage of autologous mucosal grafts likely relates to their structural compatibility with sinonasal mucosa, promoting rapid re-epithelialization and reducing fibrosis. The rapid integration and viability of the free septal mucosal graft observed in our study align with the known biological behavior of autologous endonasal transfers. Multi-layer autologous tissue structures have demonstrated favorable long-term mucosalization and structural stability when placed in contact with denuded sinus walls, effectively creating a physiological barrier against abnormal cicatricial cross-healing^15^. Similar principles have been successfully applied in skull base reconstruction, where septal mucosal grafts have demonstrated reliable mucosal regeneration^8,10^.

Importantly, no major complications, donor site morbidity, or graft-related adverse events were observed. However, several limitations should be acknowledged. These include the absence of a control group, the potential confounding effect of dexamethasone-soaked gel foam, the lack of objective assessment of patient compliance, and the relatively short follow-up period of six months. In addition, the generalizability of this technique in cases of bilateral disease is yet to be analyzed.

Future studies should include randomized controlled trials comparing this technique with established preventive strategies, as well as longer follow-up periods to assess long-term outcomes and durability.

## Conclusion

Free septal mucosal grafting appears to be a feasible technique associated with favorable postoperative outcomes for reducing middle meatal synechiae following ESS. While the results are promising, further controlled studies are required to determine its comparative effectiveness.

## Supplementary Information

Below is the link to the electronic supplementary material.

Supplementary Material 1

## Data Availability

The data sets used and/or analyzed during the current study are available from the corresponding author on reasonable request.
